# Draft *Turicibacter* sp. genome isolated from a spore-forming community in mice

**DOI:** 10.1128/mra.00385-24

**Published:** 2024-07-05

**Authors:** Kendra A. Klag, Allison M. Weis, W. Zac Stephens, June L. Round

**Affiliations:** 1 Department of Pathology, Division of Microbiology and Immunology, University of Utah School of Medicine, Salt Lake City, Utah, USA; Wellesley College, Wellesley, Massachusetts, USA

**Keywords:** gut microbiota, bacteria, metabolism, spore forming bacteria, genomics

## Abstract

*Turicibacter* is a common mammalian gut commensal; however, very few genomes have been sequenced, and little is understood regarding its importance for host health. Here, we add a complete *Turicibacter* sp. genome isolated from a spore-forming community in mice.

## ANNOUNCEMENT

*Turicibacter* is a genus of Bacilli within phylum Bacillota (1). This Gram-positive spore-forming bacterium lives in the intestines of humans and other animals (2 – 4). Recent studies point to important roles for *Turicibacter* in intestinal health (5). *Turicibacter* has two established species: *Turicibacter sanguinis*, isolated from the blood of a febrile patient (3), and *Turicibacter bilis,* isolated from chicken eggs and pig ileum (4). Other species of *Turicibacter* likely exist and have been vastly understudied.

We present a draft *Turicibacter* genome, strain KK003, isolated from mice from a spore-forming (SF) community. To isolate SF bacteria, feces from C57BL/6 mice (IACUC Protocol 00001562) were incubated anaerobically with 0.1% cysteine and 3% chloroform at 37°C for 1 h to kill vegetative bacteria. Chloroform was removed by bubbling CO_2_ through the sample for 30 s. To propagate SF, the sample was gavaged into a breeder pair of germ-free C57BL/6 mice. To isolate *Turicibacter,* feces from SF animals were chloroform treated again, serial diluted, and plated on Schaedler agar (Thermo Scientific) at 37°C anaerobically. Individual colonies were picked after 48 h, streaked to isolation, and DNA was extracted from overnight cultures in Schaedler broth using a Purelink Microbiome DNA purification kit according the manufacturer (Invitrogen) for all sequencing. One of the colonies picked was identified as *Turicibacter* by Sanger 16S rRNA gene sequencing using primers 16S_F:AGAGTTTGATCMTGGC and 16S_R:TACCTTGTTACGACTT and the Silva classifier (6).

The KK003 genome was sequenced using Illumina NovaSeq (paired-end 150) and Oxford Nanopore Technologies (ONT) minION reads. Illumina libraries were prepared with NEBNext Ultra II FS DNA kit (NEB, E7805S), and the resulting 8M reads were adapter and quality-trimmed with cutadapt (v2.10) (7) in the trim_galore (v0.6.6) wrapper using default parameters. ONT libraries were prepared without DNA shearing or size selection using rapid barcoding kit R9.4.1 chemistry (ONT, SQK-RBK004). The resulting 41.8K long reads (120.7M total bases) were basecalled, demultiplexed, adapter, barcode-trimmed with guppy (v6.0.1_gpu), quality-filtered with NanoFilt v2.8 (8) for a minimum average read-quality of 10 and minimum length of 200. The genome was assembled using SPAdes v3.15.5 within Unicycler v0.5.0 “normal mode” (9, 10), annotated with PGAP v6.6 (11). The KK003 assembly contains 2,503,176 bp, 37% GC content, and 2,447 predicted genes, which are 66.23% complete and 100th percentile by Genbank standards using CheckM v1.2.2 (12). The single contig was circularized and rotated within Unicycler by identification of linked ends and a DnaA gene that was put on the forward strand.

The taxonomic classification KK003 is genus *Turicibacter*, species unclassified (GTDB-Tk - v1.7.0). KK003 is not a member of *T. sanguinis* or *T. bilis* because it only shares 80% sequence identity with either species (using FastANI v0.1.3) (13). The nearest genome is *Turicibacter* sp. TS3 (GCF_009935875.1), which shares 99% sequence identity, indicating that *Turicibacter* should be their own species. The KK003 genome contains *bsh,* which codes for choloylglycine hydrolase, the enzyme for bile salt metabolism (5). KK003 contains a bile salt transporter, sulfur metabolism genes, sporulation genes, and elements of a Type II secretion system (Table 1).

**TABLE 1:** Key *Turicibacter* sp. K003 genomic elements

Genes	Type	Protein name and function
*bsh*	Bile salt metabolism	Choloylglycine hydrolase 1
*bsh*	Bile salt metabolism	Choloylglycine hydrolase 2
*unnamed*	Bile salt metabolism	Conjugated bile salt MFS transporter
*unnamed*	Metabolism	Putative mucin/carbohydrate-binding domain-containing protein
*unnamed*	Metabolism	Aminotransferase class I/II-fold pyridoxal phosphate-dependent enzyme
*unnamed*	Metabolism	DegT/DnrJ/EryC1/StrS family aminotransferase
*unnamed*	Sulfur metabolism	Desulfoferrodoxin family protein
*sufB*	Sulfur metabolism	sufB Fe-S cluster assembly protein
*sufC*	Sulfur metabolism	sufC Fe-S cluster assembly ATPase
*sufD*	Sulfur metabolism	sufD Fe-S cluster assembly protein
*unnamed*	Sulfur metabolism	TauE/SafE family protein sulfite exporter
*unnamed*	Sulfur metabolism	Radical SAM/SPASM domain-containing protein
*cotE*	Sporulation	CotE outer spore coat protein
*gerQ*	Sporulation	GerQ spore coat protein
*spo0A*	Sporulation	Spo0A sporulation transcription factor
*ffh*	Secretion system	Signal recognition particle protein
*ftsY*	Secretion system	FtsY signal recognition particle-docking protein
*IspA*	Secretion system	Signal peptidase II
*lepB*	Secretion system	Signal peptidase I
*unnamed*	Secretion system	Type II secretion system F family protein
*unnamed*	Secretion system	GspE/PulE family protein
*yajC*	Secretion system	YajC preprotein translocase subunit
*yihY*	Virulence	YihY/virulence factor BrkB family protein
*unnamed*	Toxin–antitoxin	Type II system antitoxin SocA domain-containing protein
*unnamed*	Toxin–antitoxin	Type II system PemK/MazF family toxin

## Data Availability

The genome is under GCF_037014675.1; reads are SRR28014011, SRR28014012.
